# Evaluating the Maximal Violation of the Original Bell Inequality by Two-Qudit States Exhibiting Perfect Correlations/Anticorrelations

**DOI:** 10.3390/e20110829

**Published:** 2018-10-29

**Authors:** Andrei Y. Khrennikov, Elena R. Loubenets

**Affiliations:** 1 International Center for Mathematical Modeling, Linnaeus University, 35195 Vaxjo, Sweden; 2 Applied Mathematics Department, National Research University Higher School of Economics, 101000 Moscow, Russia

**Keywords:** original Bell inequality, perfect correlation/anticorrelation, qudit states, quantum bound, measure of classicality

## Abstract

We introduce the general class of symmetric two-qubit states guaranteeing the perfect correlation or anticorrelation of Alice and Bob outcomes whenever some spin observable is measured at both sites. We prove that, for all states from this class, the maximal violation of the original Bell inequality is upper bounded by $\frac{3}{2}$ and specify the two-qubit states where this quantum upper bound is attained. The case of two-qutrit states is more complicated. Here, for all two-qutrit states, we obtain the same upper bound $\frac{3}{2}$ for violation of the original Bell inequality under Alice and Bob spin measurements, but we have not yet been able to show that this quantum upper bound is the least one. We discuss experimental consequences of our mathematical study.

## 1. Introduction

The recent loophole free experiments [1,2,3] demonstrated violations of classical bounds for the wide class of the Bell-type inequalities which derivations are not based on perfect (anti-) correlations, for example, the Clauser–Horne–Shimony–Holt (CHSH) inequality [4] and its further various generalizations [5,6,7,8,9,10,11,12,13,14]. These experiments have very high value for foundations of quantum mechanics (QM) and interrelation between QM and hidden variable models, see, for example, [15,16,17,18,19,20,21,22] for recent debates.

However, John Bell started his voyage beyond QM not with such inequalities, but with the original Bell inequality [23,24] the derivation of which is based on perfect anticorrelations—the condition which is explicitly related to the Einstein–Podolsky–Rosen (EPR) argument [25].

At the time of the derivation of the original Bell inequality, the experimental technology was not so advanced and preparation of sufficiently clean ensembles of singlet states was practically dificult. Therefore, Bell enthusiastically supported the proposal of Clauser, Horne, Shimony, and Holt, which is based on a new scheme (without exploring perfect correlations) and the CHSH inequality [4].

The tremendous technological success of recent years, especially, in preparation of the two-qubit singlet state and high efficiency detection, makes the original Bell’s project at least less difficult. This novel situation attracted again attention to the original Bell inequality [26]. We also point to related theoretical studies on the original Bell inequality which were done during the previous years, see [27,28,29,30,31]. In [29,31], it is, for example, shown that, unlike the CHSH inequality, the original Bell inequality distinguishes between classicality and quantum separability.

Finally, we point to a practically unknown paper of Pitowsky [32] where he claims that by violating the original Bell inequality and its generalizations it would be possible to approach a higher degree of nonclassicality than for the CHSH-like inequalities.

This claim is built upon the fact that, for the CHSH inequality $\left|B_{clas}^{\mathrm{CHSH}}\right|\leq 2,$ the fraction $F_{\mathrm{CHSH}}^{\left(\rho_{d}\right)}$ of the quantum (Tsirelson) upper bound [33,34] $2\sqrt{2}$ to the classical one is equal to $F_{\mathrm{CHSH}}^{\left(\rho_{d}\right)}=\sqrt{2}$ for a bipartite state $\rho_{d}$ of an arbitrary dimension d≥2, whereas, for the original Bell inequality, the fraction $F_{\mathrm{OB}}^{\left(\rho_{singlet}\right)}$ of the quantum upper bound for the two-qubit singlet (d=2) to the classical bound (equal to one see in Section 2) is given by [26,32]

(1) $$F_{\mathrm{OB}}^{\left(\rho_{singlet}\right)}=\frac{3}{2}>\sqrt{2}=F_{\mathrm{CHSH}}^{\left(\rho_{d}\right)},\;\;\;\forall d\geq 2.$$

The rigorous mathematical proof of the least upper bound $\frac{3}{2}$ on the violation of the original Bell inequality by the two-qubit singlet was presented in the article [26] written under the influence of Pitowsky’s paper [32]. In both papers—References [26,32], the considerations were restricted only to the two-qubit singlet case.

However, for the violation $F_{\mathrm{OB}}^{\left(\rho_{d}\right)}$ of the original Bell inequality by a two-qudit state $\rho_{d}$ exhibiting perfect correlations/anticorrelations, the CHSH inequality implies for all d≥2 the upper bound $\left(2\sqrt{2}-1\right)$ (see in Section 3) and the latter upper bound is more than the least upper bound $\frac{3}{2}$ proved [26,32] for the two-qubit singlet.

We stress that quantum nonlocality is not equivalent [35] to quantum entanglement and that larger violations of Bell inequalities can be reached [36] by states with less entanglement. Therefore, the proof [26] that, for the two-qubit singlet state (which is maximally entangled), the least upper bound on violation of the original Bell inequality is equal to $\frac{3}{2}$ does not automatically mean that $\frac{3}{2}$ is the least upper bound on violation of the original Bell inequality for all two-qubit states. Moreover, the proof of the least upper bound $\frac{3}{2}$ on violation of the original Bell inequality by the singlet state has no any consequence for quantifying violation of this inequality by a two-qudit state of an arbitrary dimension d≥2.

In the present paper, we rigorously prove that under Alice and Bob spin measurements, the least upper bound $\frac{3}{2}$ on the violation of the original Bell inequality holds for all two-qubit and all two-qutrit states exhibiting perfect correlations/anticorrelations. In the sequel to this article, we intend to prove that, quite similarly to the CHSH case where the least upper bound $\sqrt{2}$ on quantum violations holds for all dimensions d≥2, under the condition on perfect correlations/anticorrelations, the least upper bound $\frac{3}{2}$ on quantum violations of the original Bell inequality holds for all d≥2 (see in Section 6).

In Section 2 (Preliminaries), we present the condition [31] on perfect correlations or anticorrelations for joint probabilities and prove, under this condition, the validity of the original Bell inequality in the local hidden variable (LHV) frame. This general condition is true for any number of outcomes at each site and reduces to the Bell’s perfect correlation/anticorrelation condition [23] on the correlation function only in case of Alice and Bob outcomes ±1.

In Section 3, we analyse violation of the original Bell inequality by a two-qudit quantum state and show that, for all dimensions of a two-qudit state exhibiting perfect correlations/anticorrelations and any three qudit observables, the maximal violation of the original Bell inequality cannot exceed the value $(2\sqrt{2}-1).$

In Section 4, we introduce (Proposition 2) the general class of symmetric two-qubit density operators which guarantee perfect correlation or anticorrelation of Alice and Bob outcomes whenever some (the same) spin observable is measured at both sites. We prove (Theorem 1) that, for all states from this class, the maximal violation of the original Bell inequality is upper bounded by $\frac{3}{2}$ and specify the two-qubit states for which this quantum upper bound is attained.

In Section 5, we consider Alice and Bob spin measurements on two-qutrit states. This case is more complicated. Here, we are also able to prove the upper bound $\frac{3}{2}$ for all spin measurements on an arbitrary two-qutrit state, but we have not yet been able to find two-qutrit states for which this upper bound is attained. In future, we plan to study this problem as well as to consider spaces of higher dimensions.

In Section 6, we summarize the main results and stress that description of general density operators ensuring perfect correlations or anti-correlations for spin or polarization observables may simplify performance of a hypothetical experiment on violation of the original Bell inequality. In principle, experimenters need not prepare an ensemble of systems in the singlet state since, by Proposition 2 and Theorem 1, for such experiments, a variety of two-qubit states, pure and mixed, can be used and it might be easier to prepare some of such states.

## 2. Preliminaries: Derivation of the Original Bell Inequality in a General Case

Both Bell’s proofs [23,24] of the original Bell inequality in a local hidden variable (LHV) frame are essentially built up on two assumptions: a dichotomic character of Alice’s and Bob’s measurements plus the perfect correlation or anticorrelation of their outcomes for a definite pair of their local settings. Specifically, the latter assumption is abbreviated in quantum information as the condition on perfect correlations or anticorrelations.

In this section, we present the proof [31] of the original Bell inequality in the LHV frame for any numbers of Alice and Bob outcomes in [−1,1] and under the condition which is more general than the one introduced by Bell.

Consider an arbitrary bipartite correlation scenario with two measurement settings $a_{i},$ $b_{k},$ i,k=1,2, and any numbers of discrete outcomes $\lambda_{a},\lambda_{b}\in \left[-1,1\right]$ at Alice and Bob sites, respectively. This bipartite scenario is described by four joint measurements $(a_{i},b_{k}),$ i,k=1,2, with joint probability distributions $P_{\left(a_{i},b_{k}\right)}$ of outcomes in ${\left[-1,1\right]}^{2}$. Notation $P_{\left(a_{i},b_{k}\right)}\left(\lambda_{a},\lambda_{b}\right)$ means the joint probability of the event that, under a measurement $(a_{i},b_{k}),$ Alice observes an outcome $\lambda_{a}$ while Bob—an outcome $\lambda_{b}$. For the general framework on the probabilistic description of an arbitrary *N*-partite correlation scenario with any numbers of measurement settings and any spectral type of outcomes at each site, discrete or continuous, see [37].

For a joint measurement $(a_{i},b_{k}),$ we denote by

(2) $$\langle \lambda_{a_{i}}\rangle =\sum_{\lambda_{a},\lambda_{b}\in \left[-1,1\right]}\lambda_{a}P_{\left(a_{i},b_{k}\right)}\left(\lambda_{a},\lambda_{b}\right),\;\;\;\;\langle \lambda_{b_{k}}\rangle =\sum_{\lambda_{a},\lambda_{b}\in \left[-1,1\right]}\lambda_{b}P_{\left(a_{i},b_{k}\right)}\left(\lambda_{a},\lambda_{b}\right)$$

the averages of outcomes, observed by Alice and Bob, and by

(3) $$\langle \lambda_{a_{i}}\lambda_{b_{k}}\rangle =\sum_{\lambda_{a},\lambda_{b}\in \left[-1,1\right]}\lambda_{a}\lambda_{b}P_{\left(a_{i},b_{k}\right)}\left(\lambda_{a},\lambda_{b}\right)$$

the average of the product $\lambda_{a}\lambda_{b}$ of their outcomes.

Let, under a joint measurement $(a_{i},b_{k}),$ Alice and Bob outcomes satisfy the conditions that either the event

(4) $$\left\{\lambda_{a}=\lambda_{b}\right\}:=\left\{\left(\lambda_{a},\lambda_{b}\right)\in {\left[-1,1\right]}^{2}|\lambda_{a}=\lambda_{b}\right\}$$

or the event

(5) $$\left\{\lambda_{a}\;=-\lambda_{b}\neq 0\right\}:=\left\{\left(\lambda_{a},\lambda_{b}\right)\in {\left[-1,1\right]}^{2}|\lambda_{a}=-\lambda_{b}\neq 0\right\}$$

are observed with certainty, that is [31]:

(6) $$\begin{array}{ccc} P_{\left(a_{i},b_{k}\right)}(\{\lambda_{a} & = & \lambda_{b}\})=\sum_{\lambda_{a}=\lambda_{b}}P_{\left(a_{i},b_{k}\right)}\left(\lambda_{a},\lambda_{b}\right)=1 \end{array}$$

or

(7) $$\begin{array}{ccc} P_{\left(a_{i},b_{k}\right)}(\{\lambda_{a} & = & -\lambda_{b}\neq 0\})=\sum_{\lambda_{a}\;=-\lambda_{b}\neq 0}P_{\left(a_{i},b_{k}\right)}\left(\lambda_{a},\lambda_{b}\right)=1, \end{array}$$

respectively.

To demonstrate that, under conditions (6) or (7) on probabilities, outcomes of Alice and Bob are perfectly correlated or anticorrelated, consider, for example, the plus sign case (6). From (6) it follows that, for arbitrary $\lambda_{a}\neq \lambda_{b},$ the joint probability

(8) $$P_{\left(a_{i},b_{k}\right)}\left(\lambda_{a},\lambda_{b}\right){|}_{\lambda_{a}\neq \lambda_{b}}=0.$$

Hence, under a joint measurement $(a_{i},b_{k}),$ the marginal probabilities at Alice and Bob sites are given by

(9) $$\begin{array}{ccc} P_{a_{i}}\left(\lambda_{a}\right) & = & \sum_{\lambda_{b}}P_{\left(a_{i},b_{k}\right)}\left(\lambda_{a},\lambda_{b}\right)=P_{\left(a_{i},b_{k}\right)}\left(\lambda_{a},\lambda_{b}\right){|}_{\lambda_{b}=\lambda_{a}},\;\;\;\;\forall \lambda_{a},\\ P_{b_{k}}\left(\lambda_{b}\right) & = & \sum_{\lambda_{a}}P_{\left(a_{i},b_{k}\right)}\left(\lambda_{a},\lambda_{b}\right)=P_{\left(a_{i},b_{k}\right)}\left(\lambda_{a},\lambda_{b}\right){|}_{\lambda_{a}=\lambda_{b}},\;\;\;\;\forall \lambda_{b}. \end{array}$$

Therefore, under this joint measurement, at Alice and Bob sites the marginal probability distributions of observed outcomes λ∈[−1,1] coincide $P_{a_{i}}\left(\lambda \right)=P_{b_{k}}\left(\lambda \right)$ and, given, for example, that Alice observes an outcome $\lambda_{a}=\lambda_{0}$, Bob observes the outcome $\lambda_{b}=\lambda_{0}$ with certainty, i.e., the conditional probability $P_{b_{k}}\left(\lambda_{b}=\lambda_{0}|\lambda_{a}=\lambda_{0}\right)=1$, $\forall \lambda_{0}.$ Also, under condition (6), the Pearson correlation coefficient $\gamma_{cor}$, considered in statistics, is given by

(10) $$\gamma_{cor}=\frac{\sum_{\lambda_{a},\lambda_{b}}\left(\lambda_{a}-\langle \lambda_{a}\rangle \right)\left(\lambda_{b}-\langle \lambda_{b}\rangle \right)P_{\left(a_{i},b_{k}\right)}\left(\lambda_{a},\lambda_{b}\right)}{\sqrt{\sum_{\lambda_{a}}{\left(\lambda_{a}-\langle \lambda_{a}\rangle \right)}^{2}P_{a_{i}}\left(\lambda_{a}\right)}\sqrt{\sum_{\lambda_{b}}{\left(\lambda_{b}-\langle \lambda_{b}\rangle \right)}^{2}P_{b_{k}}\left(\lambda_{b}\right)}}=1.$$

Therefore, under the plus sign condition (6), Alice and Bob outcomes are perfectly correlated also in the meaning generally accepted in statistics.

The minus sign case (7) is considered quite similarly and results in the relation $P_{a_{i}}\left(\lambda \right)=P_{b_{k}}\left(-\lambda \right),$ ∀λ∈[−1,1], for marginal distributions of Alice and Bob, the relation $P_{b_{k}}\left(\lambda_{b}=-\lambda_{0}|\lambda_{a}=\lambda_{0}\right)=1$, $\forall \lambda_{0}$, for the conditional probability and the Pearson correlation coefficient $\gamma_{cor}=-1.$ All this means the perfect anticorrelation of Alice and Bob outcomes.

For a joint measurement with outcomes ±1, the general conditions (6), (7) are equivalently represented by the condition on the product expectation

(11) $$\langle \lambda_{a}\lambda_{b}\rangle =\pm 1.$$

respectively, introduced originally in Bell [23]. However, for any number of outcomes in [−1,1] at both sites, Alice and Bob outcomes may be correlated or anticorrelated in the sense of (6) or (7), respectively, but their product expectation $\langle \lambda_{a}\lambda_{b}\rangle \neq \pm 1.$

Thus, under a bipartite scenario with any number of different outcomes in [−1,1], relations (6) and (7) introduced in [31], constitute the general condition on perfect correlation or anticorrelation of outcomes observed by Alice and Bob. This general perfect correlations/anticorrelations condition reduces to the Bell one (11) only in a dichotomic case with $\lambda_{a},\lambda_{b}=\pm 1.$

Let a 2×2-setting correlation scenario with joint measurements $\left(a_{i},b_{k},\right),$ i,k=1,2 and outcomes $\lambda_{a_{i}},\lambda_{b_{k}}\in \left[-1,1\right]$ admit a local hidden variable (LHV) model for joint probabilities, for details, see Section 4 in [37], that is, all joint distributions $P_{\left(a_{i},b_{k}\right)},i,k=1,2,$ admit the representation

(12) $$P_{\left(a_{i},b_{k}\right)}\left(\lambda_{a},\lambda_{b}\right)=\int_{\Omega }P_{a_{i}}\left(\lambda_{a}|\omega \right)P_{b_{k}}\left(\lambda_{b}|\omega \right)\;\nu \left(d\omega \right),\;\;\;\forall \lambda_{a_{i}},\lambda_{b_{k}},$$

via a single probability distribution ν of some variables ω∈Ω and conditional probability distributions $P_{a_{i}}(\cdot$|ω), $P_{b_{k}}(\cdot$|ω) of outcomes at Alice’s and Bob’s sites. The latter conditional probabilities are usually referred to as “local” in the sense that each of them depends only on a measurement setting at the corresponding site.

Then all scenario product expectations $\langle \lambda_{a_{i}}\lambda_{b_{k}}\rangle ,$ i,k=1,2, admit the LHV representation

(13) $$\langle \lambda_{a_{i}}\lambda_{b_{k}}\rangle =\int_{\Omega }f_{a_{i}}\left(\omega \right)\;f_{b_{k}}\left(\omega \right)\;\nu \left(d\omega \right)$$

with

(14) $$f_{a_{i}}\left(\omega \right):=\sum_{\lambda_{a}\in \left[-1,1\right]}\lambda_{a}P_{a_{i}}\left(\lambda_{a}|\omega \right)\in \left[-1,1\right],\;\;\;f_{b_{k}}\left(\omega \right):=\sum_{\lambda_{b}\in \left[-1,1\right]}\lambda_{b}P_{b_{k}}\left(\lambda_{b}|\omega \right)\in \left[-1,1\right].$$

If an LHV model (12) for joint probabilities is deterministic [37,38], then the values of functions $f_{a_{i}},$ $f_{b_{k}},$ i,k=1,2, constitute outcomes under Alice and Bob corresponding measurements with settings $a_{i}$ and $b_{k},$ respectively. However, in a stochastic LHV model [37,38], functions $f_{a_{i}},$ $f_{b_{k}}$ may take any values in [−1,1] even in a dichotomic case.

On the other side, if, for a scenario admitting an LHV model (12) and having outcomes $\lambda_{a_{i}},\lambda_{b_{k}}=\pm 1$, the Bell perfect correlation/anticorrelation restriction $\langle \lambda_{a_{i_{0}}}\lambda_{b_{k_{0}}}\rangle =\pm 1$ is fulfilled under some joint measurement $(a_{i_{0}}$, $b_{k_{0}}),$ then, in this LHV model, the corresponding functions $f_{a_{i_{0}}},f_{b_{k_{0}}}$ take only two values ±1 and, moreover, $f_{a_{i_{0}}}\left(\omega \right)=\pm f_{b_{k_{0}}}\left(\omega \right),$ ν-almost everywhere (a.e.) on Ω.

We have the following statement [31] (see Appendix, for the proof).

**Proposition** **1.** *Let, under a 2×2-setting correlation scenario with joint measurements $\left(a_{i},b_{k},\right),$i,k=1,2 and any number of outcomes $\lambda_{a_{i}},\lambda_{b_{k}}$ in [−1,1], Alice’s and Bob’s outcomes under the joint measurement $\left(a_{2},b_{1}\right)$ be perfectly correlated or anticorrelated:*

(15) $$\begin{array}{ccc} P_{\left(a_{2},b_{1}\right)}(\{\lambda_{a} & = & \lambda_{b}\})=1 \end{array}$$

*or*

(16) $$\begin{array}{ccc} P_{\left(a_{2},b_{1}\right)}(\{\lambda_{a} & = & -\lambda_{b}\neq 0\})=1 \end{array}$$

*If this scenario admits an LHV model (12), then its product expectations satisfy the original Bell inequality:*

(17) $$\left|\;\langle \lambda_{a_{1}}\lambda_{b_{1}}\rangle -\langle \lambda_{a_{1}}\lambda_{b_{2}}\rangle \right|\pm \langle \lambda_{a_{2}}\lambda_{b_{2}}\rangle \leq 1,$$

*in its perfect correlation (plus sign) or perfect anticorrelation (minus sign) forms, respectively.*

We stress that, for the validity of the original Bell inequality (17) in the LHV frame, it is suffice for condition (15) or condition (16) on perfect correlations or anticorrelations be fulfilled only under a joint measurement $\left(a_{2},b_{1}\right)$.

Furthermore, it was proved in [31] that, in the LHV frame, the original Bell inequality (17) holds under the LHV condition which is more general than conditions (15), (16) on perfect correlation/anticorrelations, does not imply for the LHV functions (14) relations $f_{a_{2}}\left(\omega \right)=\pm f_{b_{1}}\left(\omega \right),$ ν-a.e. on Ω and incorporates conditions (15), (16) on perfect correlation/anticorrelations only as particular cases.

For many bipartite quantum states admitting 2×2-setting LHV models, specifically, this general sufficient condition in [31] ensures [30,31,39] the validity of the perfect correlation form of the original Bell inequality for Alice and Bob measurements for any three qudit quantum observables $X_{a_{1}},X_{a_{2}}=X_{b_{1}},X_{b_{2}}$ with operator norms ≤1. Satisfying the perfect correlation form of the original Bell inequality (17), these states do not need to exhibit perfect correlations and may even have a negative correlation function (see relation (61) in [31]) whenever the same quantum observable $X_{a_{2}}=X_{b_{1}}$ is measured at both sites.

For example, all two-qudit Werner state [35]

(18) $$W_{d,\Phi }=\frac{1+\Phi }{2}\frac{P_{d}^{\left(+\right)}}{r_{d}^{\left(+\right)}}+\frac{1-\Phi }{2}\frac{P_{d}^{\left(-\right)}}{r_{d}^{\left(-\right)}},\;\;\;\;\Phi \in \left[-1,1\right],$$

on $C^{d}⊗C^{d},$ d≥3, separable (Φ∈[0,1]) or nonseparable (Φ∈[−1,0)), and all separable two-qubit Werner stated $W_{2,\Phi }\left(\Phi \right),$ Φ∈[0,1], satisfy the general sufficient condition, introduced in [31], and do not violate the perfect correlation form of the original Bell inequality (17) for any three quantum observables $X_{a_{1}},X_{a_{2}}=X_{b_{1}},X_{b_{2}}$ but do not exhibit perfect correlations whenever the same observable $X_{a_{2}}=X_{b_{1}}$ is measured at both sites. In (18), $P_{d}^{\left(\pm \right)}$ are the orthogonal projections onto the symmetric and antisymmetric subspaces of $C^{d}⊗C^{d}$ with dimensions $r_{d}^{\left(\pm \right)}=\mathrm{tr}\left[P_{d}^{\left(\pm \right)}\right]$ = $\frac{d(d\pm 1)}{2},$ respectively.

## 3. Quantum Violation

Consider Alice and Bob projective measurements of quantum qudit observable $X_{a_{1}},X_{a_{2}}=X_{b_{1}},$ $X_{b_{2}}$ in an arbitrary two-qudit state ρ on $C^{d}⊗C^{d}$.

In this case, Alice and Bob outcomes coincide with eigenvalues $\lambda_{a},\lambda_{b}$ of these observables and restriction $\lambda_{a},\lambda_{b}\in \left[-1,1\right]$ implies the restriction on operators norms $∥X_{a_{i}}∥,∥X_{b_{k}}∥\leq 1$. The joint probability $P_{\left(a_{i},b_{k}\right)}\left(\lambda_{a},\lambda_{b}\right)$ that, under a joint measurement $(a_{i},b_{k}),$ Alice observes an outcome $\lambda_{a},$ while Bob—and outcome $\lambda_{b}$ is given by

(19) $$\mathrm{tr}[\rho \left\{P_{X_{a_{i}}}\left(\lambda_{a}\right)⊗P_{X_{b_{k}}}\left(\lambda_{b}\right)\right\}]$$

where $P_{X_{a_{i}}}\left(\lambda_{a}\right),$ $P_{X_{b_{k}}}\left(\lambda_{b}\right)$, i,k=1,2, are the spectral projections of observables $X_{a_{i}}$ and $X_{b_{k}},$ corresponding to eigenvalues $\lambda_{a}$ and $\lambda_{b}$, respectively. The averages in (2), (3) take the form

(20) $$\langle \lambda_{a_{i}}\rangle =\mathrm{tr}\left[\rho X_{a_{i}}\right],\;\;\;\;\langle \lambda_{b_{k}}\rangle =\mathrm{tr}\left[\rho X_{b_{k}}\right],\;\;\;\;\;\langle \lambda_{a_{i}}\lambda_{b_{k}}\rangle =\mathrm{tr}\left[\rho \left\{X_{a_{i}}⊗X_{b_{k}}\right\}\right],\;i,k=1,2$$

The general conditions (15), (16) on perfect correlations or anticorrelations of Alice and Bob outcomes under a joint measurement $\left(a_{2},b_{1}\right)$ reduce to

(21) $$\begin{array}{ccc} \sum_{\lambda_{a}=\lambda_{b}}\mathrm{tr}\left[\rho \left\{P_{X_{b_{1}}}\left(\lambda_{a}\right)⊗P_{X_{b_{1}}}\left(\lambda_{b}\right)\right\}\right] & = & 1, \end{array}$$

(22) $$\begin{array}{ccc} \sum_{\lambda_{a}=-\lambda_{b}\neq 0}\mathrm{tr}\left[\rho \left\{P_{X_{b_{1}}}\left(\lambda_{a}\right)⊗P_{X_{b_{1}}}\left(\lambda_{b}\right)\right\}\right] & = & 1, \end{array}$$

respectively, and for observables with eigenvalues ±1, these conditions are equivalent to

(23) $$\mathrm{tr}[\rho \left\{X_{b_{1}}⊗X_{b_{1}}\right\}]=\pm 1.$$

Thus, under the considered quantum scenario, the left hand-side $W_{\rho_{d}}^{\left(\pm \right)}$ of the original Bell inequality (17) takes the form

(24) $$W_{\rho }^{\left(\pm \right)}\left(X_{a},X_{b_{1}},X_{b_{2}}\right)=\left|\;\mathrm{tr}\left[\rho \left\{X_{a}⊗X_{b_{1}}\right\}\right]-\mathrm{tr}\left[\rho \left\{X_{a}⊗X_{b_{2}}\right\}\right]\;\right|\pm \mathrm{tr}\left[\rho \left\{X_{b_{1}}⊗X_{b_{2}}\right\}\right],$$

where, for short, we changed the index notation $a_{1}\to a,$ and the general condition on perfect correlations/anticorrelations of Alice and Bob outcomes under a joint measurement $\left(b_{1},b_{1}\right)$ is given by (21)/(22).

It is, however, well known that the two-qubit singlet state $\rho_{singlet}$ satisfies the perfect anticorrelation (minus sign) condition (in the form (23)) whenever the same qubit observable $X_{b}$ with eigenvalues ±1 is measured at both sites but, depending on a choice of qubit observables $X_{a},X_{b_{1}},X_{b_{2}}$, this state may, however, violate [23,24] the perfect anticorrelation form of the original Bell inequality (17).

As it has been proven in [26,32], for the singlet $\rho_{singlet}$, the maximal value of the left hand-side (24) of the original Bell inequality (17) over qubit observables with eigenvalues ±1 is equal to $\frac{3}{2}$.

This value is beyond the well-known Tsirelson [33,34] maximal value $\sqrt{2}$ for the quantum violation parameter $\left|B_{quant}^{\mathrm{CHSH}}\right|/\left|B_{lhv}^{\mathrm{CHSH}}\right|$ of the Clauser–Horne–Shimony–Holt (CHSH) inequality [4] $\left|B_{lhv}^{CHSH}\right|\leq 2$ and, moreover, beyond the least upper bound $\sqrt{2}$ on the quantum violation parameter $\left|B_{quant}\right|/\left|B_{lhv}\right|$ for all unconditional Bell functionals B(·) for two settings and two outcomes per site [40,41,42,43].

On the other side, the Tsirelson bound $2\sqrt{2}$ on the quantum violation of the CHSH inequality [4] holds for a bipartite quantum state of an arbitrary dimension. For different choices of signs, this implies

(25) $$\begin{array}{ccc} \mathrm{tr}\left[\rho \left\{X_{a}⊗X_{b_{1}}\right\}\right]-\mathrm{tr}[\rho \left\{X_{a}⊗X_{b_{2}}\right\}+\mathrm{tr}[\rho \left\{X_{b_{1}}⊗X_{b_{1}}\right\}+\mathrm{tr}\left[\rho \left\{X_{b_{1}}⊗X_{b_{2}}\right\}\right] & \leq & 2\sqrt{2}\\ \mathrm{tr}\left[\rho \left\{X_{a}⊗X_{b_{1}}\right\}\right]-\mathrm{tr}[\rho \left\{X_{a}⊗X_{b_{2}}\right\}-\mathrm{tr}[\rho \left\{X_{b_{1}}⊗X_{b_{1}}\right\}-\mathrm{tr}\left[\rho \left\{X_{b_{1}}⊗X_{b_{2}}\right\}\right] & \leq & 2\sqrt{2}\\ -\mathrm{tr}\left[\rho \left\{X_{a}⊗X_{b_{1}}\right\}\right]+\mathrm{tr}[\rho \left\{X_{a}⊗X_{b_{2}}\right\}+\mathrm{tr}[\rho \left\{X_{b_{1}}⊗X_{b_{1}}\right\}+\mathrm{tr}\left[\rho \left\{X_{b_{1}}⊗X_{b_{2}}\right\}\right] & \leq & 2\sqrt{2}\\ -\mathrm{tr}\left[\rho \left\{X_{a}⊗X_{b_{1}}\right\}\right]+\mathrm{tr}[\rho \left\{X_{a}⊗X_{b_{2}}\right\}-\mathrm{tr}[\rho \left\{X_{b_{1}}⊗X_{b_{1}}\right\}-\mathrm{tr}\left[\rho \left\{X_{b_{1}}⊗X_{b_{2}}\right\}\right] & \leq & 2\sqrt{2} \end{array}$$

Combining the first line with the third one, for a two-qudit state exhibiting perfect correlations (condition (21)), we get the following upper bound

(26) $$\begin{array}{ccc} \;W_{\rho }^{\left(+\right)}\left(X_{a},X_{b_{1}},X_{b_{2}}\right){|}_{perfect} & = & \left|\;\mathrm{tr}\left[\rho \left\{X_{a}⊗X_{b_{1}}\right\}\right]-\mathrm{tr}[\rho \left\{X_{a}⊗X_{b_{2}}\right\}\right|+\mathrm{tr}\left[\rho \left\{X_{b_{1}}⊗X_{b_{2}}\right\}\right]\\ & \leq & 2\sqrt{2}-\left|\;\mathrm{tr}[\rho \left\{X_{b_{1}}⊗X_{b_{1}}\right\}]\right| \end{array}$$

on the left-hand side of the original Bell inequality. Similarly, combining the second line with the fourth one under condition (22) on perfect anticorrelations, we derive

(27) $$\begin{array}{ccc} \;W_{\rho }^{\left(-\right)}\left(X_{a},X_{b_{1}},X_{b_{2}}\right){|}_{perfect} & = & \left|\;\mathrm{tr}\left[\rho \left\{X_{a}⊗X_{b_{1}}\right\}\right]-\mathrm{tr}[\rho \left\{X_{a}⊗X_{b_{2}}\right\}\right|-\mathrm{tr}\left[\rho \left\{X_{b_{1}}⊗X_{b_{2}}\right\}\right]\\ & \leq & 2\sqrt{2}-\left|\;\mathrm{tr}[\rho \left\{X_{b_{1}}⊗X_{b_{1}}\right\}]\right| \end{array}$$

Thus, for an arbitrary two-qudit state exhibiting perfect correlation/anticorrelations whenever the same quantum observable $X_{b_{1}}$ is measured at both sites we have

(28) $$\begin{array}{ccc} \;W_{\rho }^{\left(\pm \right)}\left(X_{a},X_{b_{1}},X_{b_{2}}\right){|}_{perfect} & = & \left|\;\mathrm{tr}\left[\rho \left\{X_{a}⊗X_{b_{1}}\right\}\right]-\mathrm{tr}\left[\rho \left\{X_{a}⊗X_{b_{2}}\right\}\right]\;\right|\pm \mathrm{tr}\left[\rho \left\{X_{b_{1}}⊗X_{b_{2}}\right\}\right]\\ & \leq & 2\sqrt{2}-\left|\;\mathrm{tr}[\rho \left\{X_{b_{1}}⊗X_{b_{1}}\right\}]\right| \end{array}$$

If observable $X_{b_{1}}$ has only eigenvalues ±1, then conditions (21), (22) reduce to the Bell condition (23) and the upper bound (28) takes the form

(29) $$W_{\rho }^{\left(\pm \right)}\left(X_{a},X_{b_{1}},X_{b_{2}}\right){|}_{perfect}\leq 2\sqrt{2}-1$$

and holds for a two-qudit state ρ of an arbitrary dimension d≥2. For d=2, this upper bound is more than the maximal value $\frac{3}{2}$ proved [26,32] for the two-qubit singlet.

Therefore, in the following section, we proceed to analyze the maximal value which the left-hand of $W_{\rho }^{\left(\pm \right)}\left(X_{a},X_{b_{1}},X_{b_{2}}\right){|}_{\mathrm{perfect}}$ over all qubit observables $X_{a},X_{b_{1}},X_{b_{2}}$ with eigenvalues ±1 and all two-qubit states ρ, satisfying the perfect correlation/anticorrelation condition (23).

## 4. Two-Qubit Case

Consider the violation of the original Bell inequality (17) by a two-qubit state exhibiting perfect correlations/anticorrelations whenever the same qubit quantum observable with eigenvalues ±1 is projectively measured at both sites.

We further consider only symmetric two-qubit states ρ (identical quantum particles), that is, states on $C^{2}⊗C^{2}$ which do not change under the permutation of the Hilbert spaces $C^{2}$ in the tensor product $C^{2}⊗C^{2},$ and, for simplicity, change index notations $b_{1}\to r,b_{2}\to c$ in (24).

For d=2, a generic qubit observable *X* on $C^{2}$ admits the representation

(30) $$\begin{array}{ccc} X & = & \alpha I_{C^{2}}+r\cdot \sigma ,\;\;\;\; \end{array}$$

(31) $$\begin{array}{ccc} r\cdot \sigma & = & r_{1}\sigma_{1}+r_{2}\sigma_{2}+r_{3}\sigma_{3} \end{array}$$

where $\alpha =\frac{1}{2}\mathrm{tr}\left[X\right],$ $r=(r_{1},r_{2},r_{3})$ is a vector in $R^{3}$ with components

(32) $$r_{1}=\frac{1}{2}\mathrm{tr}\left[X\sigma_{1}\right],\;\;\;r_{2}=\frac{1}{2}\mathrm{tr}\left[X\sigma_{2}\right],\;\;\;r_{3}=\frac{1}{2}\mathrm{tr}\left[X\sigma_{3}\right],$$

and

(33) $$\sigma_{1}=|e_{1}\rangle \langle e_{2}\left|\;+\;\right|e_{2}\rangle \langle e_{1}|,\;\;\;\;\sigma_{2}=i(|e_{2}\rangle \langle e_{1}\left|\;-\right|e_{1}\rangle \langle e_{2}|),\;\;\;\sigma_{3}=|e_{1}\rangle \langle e_{1}\left|\;-\;\right|e_{2}\rangle \langle e_{2}|$$

are self-adjoint operators on $C^{2}$ with eigenvalues ±1, represented in the standard orthonormal basis $\left\{e_{1},e_{2}\right\}$ in $C^{2}$ by the Pauli matrices

(34) $$\sigma_{1}=\left(\begin{array}{cc} 0 & 1\\ 1 & 0 \end{array}\right),\;\sigma_{2}=\left(\begin{array}{cc} 0 & -i\\ i & 0 \end{array}\right),\;\sigma_{3}=\left(\begin{array}{cc} 1 & 0\\ 0 & -1 \end{array}\right).$$

Every qubit observable with eigenvalues ±1 is represented in (30) by some unit vector $∥r∥=1$ and constitutes projection $\sigma_{r}:=r\cdot \sigma$ of the qubit spin along a unit vector (direction) *r* in $R^{3}.$

Therefore, for Alice and Bob measurements of qubit observables with eigenvalues ±1, the left-hand side (24) of the original Bell inequality takes the form

(35) $$W_{\rho }^{\left(\pm \right)}\left(\sigma_{a},\sigma_{r},\sigma_{c}\right)=\left|\;\mathrm{tr}\left[\rho \left\{\sigma_{a}⊗\sigma_{r}\right\}\right]-\mathrm{tr}\left[\rho \left\{\sigma_{a}⊗\sigma_{c}\right\}\right]\;\right|\pm \mathrm{tr}\left[\rho \left\{\sigma_{r}⊗\sigma_{c}\right\}\right]$$

where a,r,c are unit vectors in $R^{3}$ and the relation

(36) $$\mathrm{tr}[\rho \left\{\sigma_{r}⊗\sigma_{r}\right\}]=\pm 1$$

constitutes the perfect correlation/anticorrelation of Alice and Bob outcomes whenever the same spin observable $\sigma_{r}$—the projection of qubit spin along the same direction *r* in $R^{3}$—is measured at both sites.

Substituting representation (31) into (35) and (36), we rewrite these relations via scalar products of vectors in $R^{3}:$

(37) $$\begin{array}{ccc} W_{\rho }^{\left(\pm \right)}\left(\sigma_{a},\sigma_{r},\sigma_{c}\right) & = & \left|\left(a,T^{\left(\rho \right)}r\right)-\left(a,T^{\left(\rho \right)}c\right)\right|\pm \left(r,T^{\left(\rho \right)}c\right), \end{array}$$

(38) $$\begin{array}{ccc} \left(r,T^{\left(\rho \right)}r\right) & = & \pm 1, \end{array}$$

where $\left(a,T^{\left(\rho \right)}r\right):=\sum_{i,j}T_{ij}^{\left(\rho \right)}a_{i}r_{j}$ and $T^{\left(\rho \right)}$ is the linear operator on $R^{3},$ defined in the canonical basis in $R^{3}$ by the matrix with real elements

(39) $$T_{ij}^{\left(\rho \right)}:=\mathrm{tr}[\rho \left\{\sigma_{i}⊗\sigma_{j}\right\},\;\;i,j=1,2,3,$$

This correlation matrix is symmetric (since ρ is symmetric), has eigenvalues $\lambda_{m},$ m=1,2,3, where all $\left|\lambda_{m}\right|\leq 1,$ and is similar by its form to the matrix considered in [44].

Let us first analyze when an arbitrary symmetric two-qubit state ρ may satisfy condition (38). By decomposing a unit vector $r=\sum_{m}\beta_{m}v_{m}$, $\sum_{m}\beta_{m}^{2}=1,$ in the orthonormal basis $\left\{v_{j},j=1,2,3\right\}$ of eigenvectors of $T^{\left(\rho \right)},$ we rewrite condition (38) in the form

(40) $$\sum_{m}\beta_{m}^{2}\left(\lambda_{m}\mp 1\right)=0.$$

Since all eigenvalues $\left|\lambda_{m}\right|\leq 1$, relation (40) implies the following statement.

**Proposition** **2.** *A symmetric two-qubit state ρ exhibits perfect correlation/anticorrelations*

(41) $$\mathrm{tr}[\rho \left\{\sigma_{r}⊗\sigma_{r}\right\}]=\pm 1$$

*if and only if its correlation matrix $T^{\left(\rho \right)}$ has at least one eigenvalue equal to ±1, respectively. In this case:*

*(1) if only one of eigenvalues of $T^{\left(\rho \right)}$ is equal to ±1, say $\lambda_{m_{0}}=\pm 1,$ then ρ satisfies the perfect correlation/anticorrelation condition (41), respectively, only for the unit vector $r=v_{m_{0}}$;*

*(2) if $T^{\left(\rho \right)}$ has two eigenvalues equal to ±1, say $\lambda_{m_{1}},\lambda_{m_{2}}$ = ±1, then ρ satisfies the perfect correlation/anticorrelation condition (41), respectively for every unit vector $r=\beta_{m_{1}}v_{m_{1}}+\beta_{m_{2}}v_{m_{2}},$$\beta_{m_{1}}^{2}+\beta_{m_{2}}^{2}=1$ in the plane determined by the eigenvectors $\left\{v_{m_{1}},v_{m_{2}}\right\}$ of $T^{\left(\rho \right)}$;*

*(3) if all three eigenvalues of $T^{\left(\rho \right)}$ are equal to ±1, then ρ satisfies the perfect correlation/anticorrelation condition (41), respectively, for any unit vector r in $R^{3}.$*

For the two-qubit Bell states

(42) $$\phi_{\left(\pm \right)}=\frac{1}{\sqrt{2}}\left(e_{1}⊗e_{1}\pm e_{2}⊗e_{2}\right),\;\;\;\;\psi_{\left(\pm \right)}=\frac{1}{\sqrt{2}}\left(e_{1}⊗e_{2}\pm e_{2}⊗e_{1}\right),$$

we have

(43) $$\begin{array}{ccc} \;T^{\left(\phi_{+}\right)} & = & \left(\begin{array}{ccc} 1 & 0 & 0\\ 0 & -1 & 0\\ 0 & 0 & 1 \end{array}\right),\;\;\;\;T^{\left(\phi_{-}\right)}=\left(\begin{array}{ccc} -1 & 0 & 0\\ 0 & 1 & 0\\ 0 & 0 & 1 \end{array}\right)\\ T^{\left(\psi_{+}\right)} & = & \left(\begin{array}{ccc} 1 & 0 & 0\\ 0 & 1 & 0\\ 0 & 0 & -1 \end{array}\right),\;\;\;\;T^{\left(\psi_{-}\right)}=\left(\begin{array}{ccc} -1 & 0 & 0\\ 0 & -1 & 0\\ 0 & 0 & -1 \end{array}\right) \end{array}$$

and this implies.

**Corollary** **1.** *(1) The Bell state $\phi_{+}$ exhibits perfect anticorrelations under spin measurements at both sites along the coordinate axis Y and perfect correlations under spin measurements at both sites along the same arbitrary direction in the coordinate plane XZ;*

*(2) The Bell state $\phi_{-}$ exhibits perfect anticorrelations under spin measurements at both sites along the coordinate axis X and perfect correlations—under spin measurements at both sites along the same arbitrary direction in the coordinate plane YZ;*

*(3) The Bell state $\psi_{+}$ exhibits perfect anticorrelations under measurements at both sites of spin projections along the coordinate axis Z and perfect correlations—under spin measurements at both along the same arbitrary direction in the coordinate plane XY;*

*(4) The Bell state (singlet) $\psi_{-}$ exhibits perfect anticorrelations under spin measurements at both sites along the same arbitrary direction in $R^{3}.$*

Let us now analyze the maximal value of the left-hand side (37) of the original Bell inequality for a two-qubit state ρ exhibiting perfect correlations/anticorrelations (38).

Under condition $∥a∥=1,$ the maximum of $W_{\rho }^{\left(\pm \right)}\left(\sigma_{a},\sigma_{r},\sigma_{c}\right)$ over *a* is reached on the unit vector

(44) $$a=\pm \frac{T^{\left(\rho \right)}\left(r-c\right)}{∥T^{\left(\rho \right)}\left(r-c\right)∥}$$

and is given by

(45) $$∥T^{\left(\rho \right)}\left(r-c\right)∥\pm \left(r,T^{\left(\rho \right)}c\right).$$

Expanding vectors $r=\sum_{m}\beta_{m}v_{m}$, $\sum \beta_{m}^{2}=1,$ $c=\sum_{m}\gamma_{m}v_{m}$, $\sum_{m}\gamma_{m}^{2}=1,$ in terms of the orthonormal eigenvectors $\left\{v_{m}\right\}$ of $T^{\left(\rho \right)},$ we rewrite (45) in the form

(46) $$\sqrt{\sum_{m=1,2,3}\lambda_{m}^{2}{\left(\beta_{m}-\gamma_{m}\right)}^{2}}\pm \sum_{m=1,2,3}\lambda_{m}\beta_{m}\gamma_{m},$$

where, due to perfect correlations/anticorrelations condition (38), the coefficients $\beta_{m}$ are specified in Proposition 2.

Consider the maximum of expression (46) over coefficients $\gamma_{m}.$ By Proposition 2, expression (46) reduces to

(47) $$\begin{array}{cc} \; & \sqrt{\sum_{\lambda_{m}^{2}=1}{\left(\beta_{m}-\gamma_{m}\right)}^{2}+\sum_{\lambda_{m}^{2}\neq 1}\lambda_{m}^{2}\gamma_{m}{}^{2}}+\sum_{\lambda_{m}^{2}=1}\beta_{m}\gamma_{m}\\ = & \sqrt{2\left(1-\sum_{\lambda_{m}^{2}=1}\beta_{m}\gamma_{m}\right)-\sum_{\lambda_{m}^{2}\neq 1}\left(1-\lambda_{m}^{2}\right)\gamma_{m}^{2}}+\sum_{\lambda_{m}^{2}=1}\beta_{m}\gamma_{m} \end{array}$$

since $\sum_{\lambda_{m}^{2}=1}\beta_{m}^{2}=1.$ From (47) it follows that, for all choices of a direction *r*—coefficients $\beta_{m}$ in (47) specified in Proposition 2, we have

(48) $$\underset{a,c}{\sup}W_{\rho }^{\left(\pm \right)}\left(\sigma_{a},\sigma_{r},\sigma_{c}\right){|}_{\mathrm{perfect}}\leq \max_{z\in [-1,1]}\left(\sqrt{2(1-z)}+z\right)=\frac{3}{2}$$

where the upper bound $\frac{3}{2}$ is, for example, reached on every Bell state where all eigenvalues of the correlation matrices $\lambda_{m}\in \left\{-1,1\right\},m=1,2,3.$

Also, if a two-qubit state, exhibiting perfect correlations/anticorrelations (see Proposition 2), has the correlation matrix with at least two eigenvalues, say $\lambda_{m_{1}},\lambda_{m_{2}},$ with $\left|\lambda_{m_{1}}\right|,\left|\lambda_{m_{2}}\right|=1,$ then the upper bound $\frac{3}{2}$ is reached on the unit vector *c* which is in the plane of eigenvectors $v_{m_{1}},v_{m_{2}}$ corresponding to these eigenvalues (vector *r* is in this plane, see Proposition 2) and satisfies condition c·r = $\sum_{\lambda_{m}^{2}=1}\beta_{m}\gamma_{m}=\frac{1}{2},$ that is, at angle π/3 to vector r.

Thus, we have proved the following new result.

**Theorem** **1.** *Let ρ be a symmetric two-qubit states on $C^{2}⊗C^{2}$ exhibiting perfect correlations/anticorrelations whenever the same qubit observable $\sigma_{r}$ is measured at both sites. Then the maximal value of the left-hand side $W_{\rho }^{\left(\pm \right)}\left(\sigma_{a},\sigma_{r},\sigma_{c}\right)$ of the original Bell inequality is given by*

(49) $$\max_{\rho ,a,r,c}W_{\rho }^{\left(\pm \right)}\left(\sigma_{a},\sigma_{r},\sigma_{c}\right){|}_{\mathrm{perfect}}=\frac{3}{2}$$

*and is reached on symmetric two-qubit states discussed in lines after Equation (48).*

We stress that this maximal value is less than the upper bound (29) following from the CHSH inequality.

## 5. Two-Qutrit Case

Consider now the violation of the original Bell inequality under Alice and Bob spin measurements on a symmetric two-qutrit state ρ on $C^{3}⊗C^{3},$ exhibiting perfect correlations or anticorrelations.

For Alice and Bob spin measurements in a two-qutrit state ρ, the left-hand side (24) of the original Bell inequality and the condition on perfect correlations/anticorrelations take the forms

(50) $$\begin{array}{ccc} W_{\rho }^{\left(\pm \right)}\left(S_{a},S_{r},S_{c}\right) & = & \left|\;\mathrm{tr}\left[\rho \left\{S_{a}⊗S_{r}\right\}\right]-\mathrm{tr}\left[\rho \left\{S_{a}⊗S_{c}\right\}\right]\;\right|\pm \mathrm{tr}\left[\rho \left\{S_{r}⊗S_{c}\right\}\right], \end{array}$$

(51) $$\begin{array}{ccc} \mathrm{tr}[\rho \left\{S_{r}⊗S_{r}\right\}] & = & \pm 1, \end{array}$$

where a,r,c are unit vectors in $R^{3}$ and

(52) $$S_{r}=r\cdot S=r_{1}S_{1}+r_{2}S_{2}+r_{3}S_{3},\;\;\;S=\left(S_{1},S_{2},S_{3}\right),$$

is the qutrit observable with eigenvalues {1,0,−1}, describing projection of qutrit spin along a unit vector *r* in $R^{3}$.

Note that if a two-qutrit state ρ exhibits perfect correlations/anticorrelations (51) under measurements in this state at both sites of spin projection along a direction *r*, the probability of event that either Alice or Bob observe at their site the outcome λ=0 is equal to zero.

In the standard orthonormal basis $\left\{e_{1},e_{2},e_{3}\right\}$ in $C^{3}$ these operators have the following matrix representations:

(53) $$S_{1}=\frac{1}{\sqrt{2}}\left(\begin{array}{ccc} 0 & 1 & 0\\ 1 & 0 & 1\\ 0 & 1 & 0 \end{array}\right),\;\;\;S_{2}=\frac{1}{\sqrt{2}}\left(\begin{array}{ccc} 0 & -i & 0\\ i & 0 & -i\\ 0 & i & 0 \end{array}\right),\;\;\;S_{3}=\left(\begin{array}{ccc} 1 & 0 & 0\\ 0 & 0 & 0\\ 0 & 0 & -1 \end{array}\right)$$

and

(54) $$\;S_{r}=\left(\begin{array}{ccc} r_{3} & \frac{r_{1}-ir_{2}}{\sqrt{2}} & 0\\ \frac{r_{1}+ir_{2}}{\sqrt{2}} & 0 & \frac{r_{1}-ir_{2}}{\sqrt{2}}\\ 0 & \frac{r_{1}+ir_{2}}{\sqrt{2}} & -r_{3} \end{array}\right)$$

In view of (52), quite similarly to our techniques in Section 4 we introduce for a symmetric two-qutrit state ρ the correlation matrix $Z^{\left(\rho \right)}$ with real elements

(55) $$Z_{ij}^{\left(\rho \right)}=\mathrm{tr}\left[\rho \left\{S_{i}⊗S_{j}\right\}\right],$$

which is symmetric, diagonalized and has eigenvalues $\left|\lambda_{m}\right|\leq 1,$ and this allows us to rewrite (50), (51) in the form:

(56) $$\begin{array}{ccc} \;W_{\rho }^{\left(\pm \right)}\left(S_{a},S_{r},S_{c}\right) & = & \left|\left(a,Z^{\left(\rho \right)}r\right)-\left(a,Z^{\left(\rho \right)}c\right)\right|\pm \left(r,Z^{\left(\rho \right)}c\right),\\ \left(r,Z^{\left(\rho \right)}r\right) & = & \pm 1. \end{array}$$

These expressions are quite the same by their form to expressions (37), (38) for a two-qubit state. By using the same techniques as in a qubit case, we derive

(57) $$\underset{a,c}{\sup}W_{\rho }^{\left(\pm \right)}\left(S_{a},S_{r},S_{c}\right){|}_{\mathrm{perfect}}\leq \frac{3}{2}.$$

We, however, do not know whether under the considered measurements this supremum is reached.

**Theorem** **2.** *Let ρ be a symmetric two-qutrit states on $C^{3}⊗C^{3}$ exhibiting perfect correlations/anticorrelations whenever spin projection $S_{r}$ along a direction r is measured at both sites. Then, under Alice and Bob spin measurements on these two-qutrit states, the maximal value of the left-hand side $W_{\rho }^{\left(\pm \right)}\left(S_{a},S_{r},S_{c}\right)$ of the original Bell inequality (17) is upper bounded as*

(58) $$\underset{\rho ,a,r,c}{\sup}W_{\rho }^{\left(\pm \right)}\left(S_{a},S_{r},S_{c}\right){|}_{\mathrm{perfectBell}}\leq \frac{3}{2}.$$

This two-qutrit upper bound is less than the upper bound (29) following from the CHSH inequality.

## 6. Conclusions

As was pointed out in the Introduction, the recent tremendous developments in quantum technologies make experiments to test the original Bell inequality at least less difficult. This stimulates interest in novel theoretical, foundational, and mathematical studies on this inequality. In particular, it is important to find the quantum bound, the analog of the Tsirelson bound, for the original Bell inequality. It was well-known that in the two-qubit singlet case this bound equals 3/2, see, e.g., [26,32]. A year ago, I. Basieva and A. Khrennikov came with the conjecture [45] that the same upper bound holds in case of arbitrary two-qudit states and qudit observables coupled by perfect correlations/anticorrelations. The question of quantum upper bound for the original Bell inequality became actual in connection with studies on quantum-like modeling of psychological behavior, see related paper [46].

In the present article, we have proven this conjecture for all two-qubit states and all traceless qubit observables and all two-qubit states and spin qutrit observables. This is the first step towards justifying this conjecture for an arbitrary two-qudit case, and the authors of the present paper plan to continue studies on this problem. Since in the multi-dimensional case the analytical expressions are very complex, it may be useful to try to perform preliminary numerical study, cf. [47]. We also point to technique for evaluation of the quantum upper bound which was elaborated in [48,49] and tested on the CHSH-like inequalities. In principle, this technique can be applied to the original Bell inequality.

## Appendix A

Consider the proof of Proposition 1.

Let, for a joint measurement $\left(a_{2},b_{1}\right)$, the perfect anticorrelation (16) be fulfilled and this scenario admit an LHV model (12). This and (14) imply:

$$0\leq \int_{\Omega }\left|f_{a_{2}}\left(\omega \right)+f_{b_{1}}\left(\omega \right)\right|\;\nu \left(d\omega \right)$$

$$=\int_{\Omega }\left|\sum_{\lambda_{a},\lambda_{b}}\left(\lambda_{a}+\lambda_{b}\right)P_{a_{2}}\left(\lambda_{a}|\omega \right)P_{b_{1}}\left(\lambda_{b}|\omega \right)\right|\nu \left(d\omega \right)$$

$$\leq \int_{\Omega }\sum_{\lambda_{a},\lambda_{b}}\left|\lambda_{a}+\lambda_{b}\right|\;P_{a_{2}}\left(\lambda_{a}|\omega \right)P_{b_{1}}\left(\lambda_{b}|\omega \right)\nu \left(d\omega \right)\leq 2\sum_{\lambda_{a}\neq -\lambda_{b}}P_{\left(a_{2},b_{1}\right)}\left(\lambda_{a},\lambda_{b}\right)=0.$$

Thus, under condition (16) on scenario joint probabilities, the LHV functions $f_{a_{2}}\left(\omega \right)=-f_{b_{1}}\left(\omega \right),$ ν-a.e. on Ω. Quite similarly, for the case of perfect correlations (15) we derive $f_{a_{2}}\left(\omega \right)=f_{b_{1}}\left(\omega \right),$ ν-a.e. on Ω. These relations and the number inequality

$$\left|x-y\right|\leq 1-xy,\;\;\;\forall \;x,y\in \left[-1,1\right],$$

give:

$$\left|\langle \lambda_{a_{1}}\lambda_{b_{1}}\rangle -\langle \lambda_{a_{1}}\lambda_{b_{2}}\rangle \right|\pm \langle \lambda_{a_{2}}\lambda_{b_{2}}\rangle$$

$$=\left|\int_{\Omega }f_{a_{1}}\left(\omega \right)f_{b_{1}}\left(\omega \right)-f_{a_{1}}\left(\omega \right)f_{b_{2}}\left(\omega \right)\;\nu \left(d\omega \right)\right|\pm \int_{\Omega }f_{a_{2}}\left(\omega \right)f_{b_{2}}\left(\omega \right)\nu \left(d\omega \right)$$

$$\leq \int_{\Omega }\left|(f_{b_{1}}\left(\omega \right)-f_{b_{2}}\left(\omega \right))\right|\nu \left(d\omega \right)\pm \int_{\Omega }f_{a_{2}}\left(\omega \right)f_{b_{2}}\left(\omega \right)\;\nu \left(d\omega \right)\leq 1.$$

This proves the statement.
